# Tetra­kis(1,1,1-trifluoro­acetyl­acetonato-κ^2^ *O*,*O*′)zirconium(IV) toluene solvate

**DOI:** 10.1107/S1600536808014499

**Published:** 2008-05-21

**Authors:** Maryke Steyn, Andreas Roodt, Gideon Steyl

**Affiliations:** a Department of Chemistry, University of the Free State, PO Box 339, Bloemfontein 9300, South Africa

## Abstract

In the title compound, [Zr(C_5_H_4_F_3_O_2_)_4_]·C_7_H_8_, the Zr atom is in a square-anti­prismatic coordination geometry that comprises four *O*,*O*′-bidentate trifluoro­acetyl­acetonate ligands. The O—Zr—O bite angles of the acetonate ligands range from 75.27 (5) to 75.41 (5)°. The Zr atom is located on a twofold rotation axis.

## Related literature

For β-diketone complexes of zirconium, see: Allard (1976 ▶); Clegg (1987 ▶); Calderazzo *et al.* (1998 ▶); Davis & Einstein (1978 ▶); Elder (1969 ▶); Silverton & Hoard (1963 ▶). For the unsolvated title complex, see: Kurat’eva *et al.* (2007 ▶). For a comparison with the isomorphous hafnium complex, see: Viljoen *et al.* (2008 ▶).
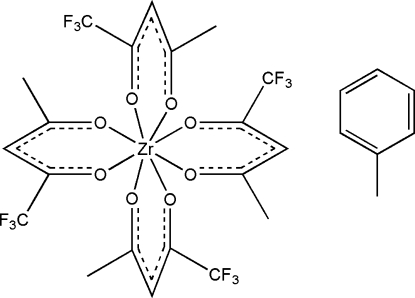


## Experimental

### 

#### Crystal data

[Zr(C_5_H_4_F_3_O_2_)_4_]·C_7_H_8_
*M* *_r_* = 887.82
Monoclinic, 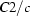

*a* = 22.537 (5) Å
*b* = 8.054 (5) Å
*c* = 22.786 (5) Å
β = 118.383 (5)°
*V* = 3639 (3) Å^3^
*Z* = 4
Mo *K*α radiation
μ = 0.41 mm^−1^
*T* = 100 (2) K
0.33 × 0.22 × 0.20 mm

#### Data collection

Bruker SMART APEXII CCD area-detector diffractometer
Absorption correction: multi-scan (*SADABS*; Bruker, 1998 ▶) *T*_min_ = 0.876, *T*_max_ = 0.922
14897 measured reflections
3975 independent reflections
3559 reflections with *I* > 2σ(*I*)
*R*_int_ = 0.029

#### Refinement

*R*[*F* ^2^ > 2σ(*F* ^2^)] = 0.029
*wR*(*F* ^2^) = 0.070
*S* = 1.05
3975 reflections
259 parameters
H atoms treated by a mixture of independent and constrained refinement
Δρ_max_ = 0.50 e Å^−3^
Δρ_min_ = −0.45 e Å^−3^

### 

Data collection: *APEX2* (Bruker, 2005 ▶); cell refinement: *SAINT-Plus* (Bruker, 2004 ▶); data reduction: *SAINT-Plus* and *XPREP* (Bruker, 2004 ▶); program(s) used to solve structure: *SHELXS97* (Sheldrick, 2008 ▶); program(s) used to refine structure: *SHELXL97* (Sheldrick, 2008 ▶); molecular graphics: *DIAMOND* (Brandenburg & Putz, 2005 ▶); software used to prepare material for publication: *SHELXL97*.

## Supplementary Material

Crystal structure: contains datablocks I, global. DOI: 10.1107/S1600536808014499/ng2456sup1.cif

Structure factors: contains datablocks I. DOI: 10.1107/S1600536808014499/ng2456Isup2.hkl

Additional supplementary materials:  crystallographic information; 3D view; checkCIF report

## Figures and Tables

**Table:** 

Zr—O01	2.1633 (13)
Zr—O11	2.1679 (13)
Zr—O02	2.1973 (15)
Zr—O12	2.2079 (15)

**Table:** 

O01^i^—Zr—O01	142.07 (7)
O01—Zr—O11	80.66 (5)
O11—Zr—O11^i^	142.56 (7)
O01—Zr—O02	75.41 (5)
O11—Zr—O02	76.85 (5)
O01—Zr—O12	76.90 (5)
O11—Zr—O12	75.27 (5)

Symmetry code: (i) 
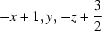
.

**Fig. 1.**
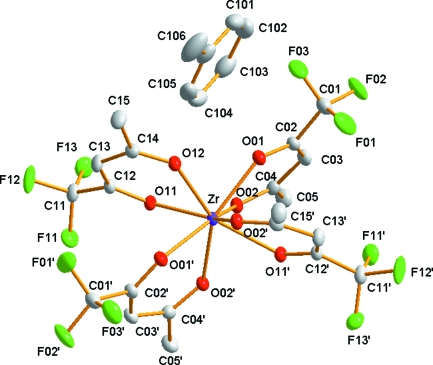
: Representation of the title compound (I), showing the numbering scheme and displacement ellipsoids (50% probability). For the carbon atoms, first digit refers to acetonato backbone moiety, second digit to atom on this backbone. Hydrogen and fluorine atoms are labeled in accordance with specific carbon attached to on acetylacetonato backbone. Hydrogen atoms are omitted for clarity.

## supplementary crystallographic information

### Comment

Zirconium complexes containing diketonato ligands have been reported in the past (Silverton & Hoard, 1963; Allard, 1976; Clegg, 1987; Elder *et al.*, 1969; Calderazzo *et al.*, 1998; Davis & Einstein, 1978). The following diketonato ligand complexes have been reported; acetylacetone (acacH), hexafluoroacetylacetone (hfaaH), tropolone (tropH) and trifluoroacetylacetone (tfaaH). Our research group's interest is in the solvated form of trifluoroacetylacetonato-zirconium(IV) complexes. The title compound is presented as an example of a toluene solvated species.

The title compound is composed of an eight-coordinate zirconium metal centre in which the four O,*O*-donating bidentate tfaa-ligands are arranged around the metal centre to give a distorted square antiprismatic geometry. The molecule has an inversion centre on the metal with two bidentate ligands on either side including a non-disordered toluene solvate molecule found in a 1:1 ratio to the zirconium complex. The bidentate ligands are coordinated in an alternating configuration with respect to the CF_3_ groups. This ligand interchange can be visualized as four fins or propellar blades around the metal centre. The distorted square antiprism is defined by the intersection of the two planes formed by the ligand-backbone (O—C—C—C—O) and the O—Zr—O bite angle, which bend inward at an angle of 19.84 - 20.23°. Within the bidentate ligand structural representation, the Zr—O_1_ (CF_3_-side bond) and Zr—O_2_ (CH_3_-side bond) bond distances are unequal, varying by 0.034 - 0.040 Å. The bite angles of the bidentate ligands to the metal centre are 75.27 (5) and 75.41 (5)°, respectively.

π-π Stacking is observed between the two toluene solvate molecules C100—C106 and C100—C106 (-1/2 + *x*, 0.5 - *y*, -1/2 + *z*) with an interplanar distance of 3.548 Å and a centroid-to-centroid distance of 4.933 Å. Weak C—H-π intermolecular interactions are observed between the toluene solvate and the tfaa-moiety: C105—H105 to C12 (3.786 Å, 172.96 °) and C106—H10D to C14 (3.702 Å, 67.74 °), respectively.

Compared to a recently published structure (Kurat'eva *et al.*, 2007) of the unsolvated complex the deviation in characteristics between the solvated and unsolvated species are minimal. The Zr—O bond length on the CF_3_-side of the acetylacetonato group, is shorter than the CH_3_-side bond by an average of 0.035 Å. The angles at which the ligands bend out of the O—Zr—O plane show the most notable difference, with the steric interaction of the toluene molecule distorting the two fins formed on the zirconium complex. This observation is further clarified by an overlay of the solvated and unsolvated zirconium complexes, which has an RMS overlay error of less than 1 Å (excluding H and CF_3_) indicating the distortion impact of the toluene solvate.

### Experimental

Chemicals were purchased from Sigma-Aldrich and used as received except for toluene which was dried by passage over alumina. Synthesis of [Zr(Tfaa)_4_] was done under Schlenk conditions. ZrCl_4_ (218.8 mg, 0.9389 mmol) was added to TfaaNa (663.4 mg, 3.768 mmol, 4eq) in dry toluene (50 ml). This slurry was refluxed for 16 h at 80°C before filtration of the remaining precipitates. The filtrate was recrystalized at -23°C to yield crystals suitable for data collection. Spectroscopic data: ^19^F NMR (C_6_D_6_, 564.77 MHz, p.p.m.): -75.3; IR (ATR) ν(CO): 1533 cm^-1^.

### Refinement

The aromatic, methine, and methyl H atoms were placed in geometrically idealized positions (C—H = 0.93–0.98) and constrained to ride on their parent atoms with *U*_iso_(H) = 1.2*U*_eq_(C) for aromatic and methine, and *U*_iso_(H) = 1.5*U*_eq_(C) for methyl protons. Torsion angles for methyl protons were refined from electron density.

### Crystal data

**Table:**

[Zr(C_5_H_4_F_3_O_2_)_4_]·C_7_H_8_	*F*_000_ = 1792
*M**_r_* = 887.82	*D*_x_ = 1.621 Mg m^−^^3^
Monoclinic, *C*2/*c*	Mo *K*α radiation λ = 0.71073 Å
Hall symbol: -C 2yc	Cell parameters from 7203 reflections
*a* = 22.537 (5) Å	θ = 2.7–28.3º
*b* = 8.054 (5) Å	µ = 0.41 mm^−^^1^
*c* = 22.786 (5) Å	*T* = 100 (2) K
β = 118.383 (5)º	Cuboid, colourless
*V* = 3639 (3) Å^3^	0.33 × 0.22 × 0.20 mm
*Z* = 4	

### Data collection

**Table:**

Bruker SMART CCD area-detector diffractometer	3975 independent reflections
Radiation source: fine-focus sealed tube	3559 reflections with *I* > 2σ(*I*)
Monochromator: graphite	*R*_int_ = 0.029
*T* = 100(2) K	θ_max_ = 27.0º
φ and ω scans	θ_min_ = 2.1º
Absorption correction: multi-scan(SADABS; Bruker, 1998)	*h* = −28→25
*T*_min_ = 0.876, *T*_max_ = 0.922	*k* = −10→7
14897 measured reflections	*l* = −28→29

### Refinement

**Table:**

Refinement on *F*^2^	Secondary atom site location: difference Fourier map
Least-squares matrix: full	Hydrogen site location: inferred from neighbouring sites
*R*[*F*^2^ > 2σ(*F*^2^)] = 0.029	H atoms treated by a mixture of independent and constrained refinement
*wR*(*F*^2^) = 0.070	*w* = 1/[σ^2^(*F*_o_^2^) + (0.0265*P*)^2^ + 6.0538*P*] where *P* = (*F*_o_^2^ + 2*F*_c_^2^)/3
*S* = 1.05	(Δ/σ)_max_ = 0.001
3975 reflections	Δρ_max_ = 0.50 e Å^−^^3^
259 parameters	Δρ_min_ = −0.45 e Å^−^^3^
Primary atom site location: structure-invariant direct methods	Extinction correction: none

### Special details

**Table:**

Geometry. All e.s.d.'s (except the e.s.d. in the dihedral angle between two l.s. planes) are estimated using the full covariance matrix. The cell e.s.d.'s are taken into account individually in the estimation of e.s.d.'s in distances, angles and torsion angles; correlations between e.s.d.'s in cell parameters are only used when they are defined by crystal symmetry. An approximate (isotropic) treatment of cell e.s.d.'s is used for estimating e.s.d.'s involving l.s. planes.
Refinement. Refinement of *F*^2^ against ALL reflections. The weighted *R*-factor *wR* and goodness of fit *S* are based on *F*^2^, conventional *R*-factors *R* are based on *F*, with *F* set to zero for negative *F*^2^. The threshold expression of *F*^2^ > σ(*F*^2^) is used only for calculating *R*-factors(gt) *etc*. and is not relevant to the choice of reflections for refinement. *R*-factors based on *F*^2^ are statistically about twice as large as those based on *F*, and *R*- factors based on ALL data will be even larger.

### Fractional atomic coordinates and isotropic or equivalent isotropic displacement parameters (Å^2^)

**Table:**

	*x*	*y*	*z*	*U*_iso_*/*U*_eq_	Occ. (<1)
Zr	0.5000	0.16714 (3)	0.7500	0.01245 (7)	
F13	0.26810 (6)	−0.05226 (14)	0.66176 (6)	0.0295 (3)	
F11	0.31909 (6)	−0.06998 (14)	0.76862 (6)	0.0287 (3)	
F03	0.51411 (7)	0.40573 (15)	0.95310 (6)	0.0340 (3)	
F02	0.45468 (7)	0.22014 (15)	0.96764 (6)	0.0368 (3)	
O11	0.39952 (6)	0.08075 (15)	0.72452 (6)	0.0158 (3)	
O02	0.51936 (6)	−0.05446 (15)	0.81277 (6)	0.0166 (3)	
O01	0.49399 (6)	0.25443 (15)	0.83681 (6)	0.0169 (3)	
F12	0.24250 (6)	0.11472 (15)	0.71971 (7)	0.0366 (3)	
O12	0.43558 (6)	0.38983 (15)	0.71261 (6)	0.0171 (3)	
F01	0.40934 (7)	0.38633 (18)	0.88399 (6)	0.0431 (4)	
C13	0.33615 (9)	0.3227 (2)	0.71690 (10)	0.0200 (4)	
C02	0.48042 (9)	0.1790 (2)	0.87851 (9)	0.0174 (4)	
C05	0.50748 (11)	−0.2801 (2)	0.87225 (10)	0.0244 (4)	
H05A	0.5085	−0.3440	0.8372	0.037*	
H05B	0.4693	−0.3129	0.8774	0.037*	
H05C	0.5481	−0.2994	0.9132	0.037*	
C12	0.34797 (9)	0.1566 (2)	0.72089 (8)	0.0160 (3)	
C01	0.46413 (10)	0.2976 (2)	0.92118 (9)	0.0230 (4)	
C11	0.29404 (9)	0.0373 (2)	0.71787 (10)	0.0214 (4)	
C14	0.38103 (9)	0.4351 (2)	0.70992 (9)	0.0182 (4)	
C03	0.47956 (10)	0.0124 (2)	0.88819 (9)	0.0200 (4)	
C04	0.50208 (9)	−0.1001 (2)	0.85506 (9)	0.0174 (4)	
C102	0.67683 (12)	0.0133 (3)	1.07100 (11)	0.0402 (6)	
H102	0.6803	−0.0134	1.1122	0.048*	
C15	0.36143 (10)	0.6138 (2)	0.69579 (11)	0.0257 (4)	
H15A	0.4003	0.6786	0.7036	0.039*	
H15B	0.3440	0.6517	0.7246	0.039*	
H15C	0.3274	0.6260	0.6501	0.039*	
C105	0.66546 (11)	0.0938 (4)	0.94967 (11)	0.0403 (6)	
H105	0.6612	0.1195	0.9080	0.048*	
C104	0.67351 (12)	−0.0692 (3)	0.96984 (12)	0.0428 (6)	
H104	0.6748	−0.1519	0.9420	0.051*	
C100	0.66358 (10)	0.2198 (3)	0.98939 (11)	0.0337 (5)	
C101	0.66888 (11)	0.1768 (3)	1.05052 (11)	0.0352 (5)	
H101	0.6671	0.2593	1.0782	0.042*	
C103	0.67966 (12)	−0.1103 (3)	1.03094 (13)	0.0411 (6)	
H103	0.6857	−0.2203	1.0450	0.049*	
C106	0.65652 (13)	0.3975 (4)	0.96634 (16)	0.0586 (8)	
H10A	0.6561	0.4691	0.9998	0.088*	0.50
H10B	0.6151	0.4103	0.9255	0.088*	0.50
H10C	0.6938	0.4262	0.9590	0.088*	0.50
H10D	0.6540	0.4013	0.9231	0.088*	0.50
H10E	0.6949	0.4601	0.9974	0.088*	0.50
H10F	0.6162	0.4442	0.9639	0.088*	0.50
H03	0.4677 (11)	−0.027 (3)	0.9208 (10)	0.024 (6)*	
H13	0.2975 (12)	0.361 (3)	0.7144 (11)	0.028 (6)*	

### Atomic displacement parameters (Å^2^)

**Table:**

	*U*^11^	*U*^22^	*U*^33^	*U*^12^	*U*^13^	*U*^23^
Zr	0.01529 (12)	0.00880 (12)	0.01714 (12)	0.000	0.01087 (9)	0.000
F13	0.0252 (6)	0.0253 (6)	0.0364 (6)	−0.0084 (5)	0.0133 (5)	−0.0067 (5)
F11	0.0325 (6)	0.0214 (6)	0.0384 (7)	−0.0030 (5)	0.0221 (6)	0.0069 (5)
F03	0.0518 (8)	0.0227 (6)	0.0398 (7)	−0.0104 (6)	0.0318 (6)	−0.0121 (5)
F02	0.0681 (9)	0.0251 (6)	0.0407 (7)	−0.0054 (6)	0.0450 (7)	−0.0025 (5)
O11	0.0164 (6)	0.0125 (6)	0.0217 (6)	0.0012 (5)	0.0116 (5)	0.0004 (5)
O02	0.0188 (6)	0.0140 (6)	0.0206 (6)	0.0007 (5)	0.0123 (5)	0.0025 (5)
O01	0.0222 (6)	0.0131 (6)	0.0199 (6)	−0.0008 (5)	0.0136 (5)	0.0003 (5)
F12	0.0295 (7)	0.0204 (6)	0.0776 (10)	0.0012 (5)	0.0399 (7)	0.0000 (6)
O12	0.0193 (6)	0.0134 (6)	0.0231 (6)	0.0016 (5)	0.0136 (5)	0.0024 (5)
F01	0.0451 (8)	0.0518 (9)	0.0367 (7)	0.0246 (7)	0.0229 (6)	−0.0001 (6)
C13	0.0184 (9)	0.0161 (9)	0.0311 (10)	0.0030 (7)	0.0163 (8)	0.0012 (8)
C02	0.0192 (9)	0.0182 (9)	0.0173 (8)	−0.0009 (7)	0.0106 (7)	−0.0008 (7)
C05	0.0343 (11)	0.0156 (9)	0.0300 (10)	−0.0006 (8)	0.0208 (9)	0.0034 (8)
C12	0.0164 (8)	0.0158 (9)	0.0192 (8)	0.0001 (7)	0.0113 (7)	−0.0005 (7)
C01	0.0319 (11)	0.0205 (10)	0.0228 (9)	0.0012 (8)	0.0180 (8)	0.0014 (7)
C11	0.0203 (9)	0.0160 (9)	0.0342 (10)	0.0017 (7)	0.0180 (8)	0.0001 (8)
C14	0.0217 (9)	0.0146 (9)	0.0202 (9)	0.0034 (7)	0.0115 (7)	0.0012 (7)
C03	0.0260 (10)	0.0186 (9)	0.0212 (9)	−0.0010 (7)	0.0159 (8)	0.0023 (7)
C04	0.0163 (9)	0.0164 (9)	0.0194 (8)	−0.0018 (7)	0.0084 (7)	0.0020 (7)
C102	0.0378 (13)	0.0551 (16)	0.0294 (11)	−0.0099 (11)	0.0174 (10)	0.0037 (11)
C15	0.0268 (10)	0.0148 (9)	0.0406 (11)	0.0043 (8)	0.0202 (9)	0.0043 (8)
C105	0.0264 (11)	0.0699 (18)	0.0234 (10)	−0.0085 (11)	0.0108 (9)	−0.0024 (11)
C104	0.0286 (12)	0.0546 (17)	0.0453 (14)	−0.0078 (11)	0.0177 (11)	−0.0252 (12)
C100	0.0176 (10)	0.0387 (13)	0.0346 (11)	−0.0053 (9)	0.0040 (9)	0.0047 (10)
C101	0.0305 (12)	0.0414 (14)	0.0330 (11)	−0.0061 (10)	0.0145 (9)	−0.0128 (10)
C103	0.0284 (12)	0.0329 (13)	0.0552 (15)	−0.0059 (10)	0.0144 (11)	−0.0016 (11)
C106	0.0288 (13)	0.0518 (17)	0.0718 (19)	−0.0054 (12)	0.0048 (13)	0.0233 (15)

### Geometric parameters (Å, °)

**Table:**

Zr—O01^i^	2.1633 (13)	C05—H05C	0.9600
Zr—O01	2.1633 (13)	C12—C11	1.525 (3)
Zr—O11	2.1679 (13)	C14—C15	1.496 (3)
Zr—O11^i^	2.1679 (13)	C03—C04	1.419 (3)
Zr—O02^i^	2.1973 (15)	C03—H03	0.95 (2)
Zr—O02	2.1973 (15)	C102—C103	1.372 (4)
Zr—O12^i^	2.2079 (15)	C102—C101	1.381 (4)
Zr—O12	2.2079 (15)	C102—H102	0.9300
F13—C11	1.336 (2)	C15—H15A	0.9600
F11—C11	1.335 (2)	C15—H15B	0.9600
F03—C01	1.333 (2)	C15—H15C	0.9600
F02—C01	1.330 (2)	C105—C104	1.374 (4)
O11—C12	1.280 (2)	C105—C100	1.374 (4)
O02—C04	1.254 (2)	C105—H105	0.9300
O01—C02	1.281 (2)	C104—C103	1.373 (4)
F12—C11	1.337 (2)	C104—H104	0.9300
O12—C14	1.256 (2)	C100—C101	1.384 (3)
F01—C01	1.326 (2)	C100—C106	1.506 (4)
C13—C12	1.359 (3)	C101—H101	0.9300
C13—C14	1.421 (3)	C103—H103	0.9300
C13—H13	0.90 (2)	C106—H10A	0.9600
C02—C03	1.362 (3)	C106—H10B	0.9600
C02—C01	1.527 (3)	C106—H10C	0.9600
C05—C04	1.492 (3)	C106—H10D	0.9600
C05—H05A	0.9600	C106—H10E	0.9600
C05—H05B	0.9600	C106—H10F	0.9600
			
O01^i^—Zr—O01	142.07 (7)	F13—C11—C12	111.10 (15)
O01^i^—Zr—O11	111.77 (5)	F12—C11—C12	112.97 (15)
O01—Zr—O11	80.66 (5)	O12—C14—C13	122.83 (16)
O01^i^—Zr—O11^i^	80.66 (5)	O12—C14—C15	118.18 (16)
O01—Zr—O11^i^	111.77 (5)	C13—C14—C15	118.94 (17)
O11—Zr—O11^i^	142.56 (7)	C02—C03—C04	120.48 (17)
O01^i^—Zr—O02^i^	75.41 (5)	C02—C03—H03	118.7 (13)
O01—Zr—O02^i^	141.24 (5)	C04—C03—H03	120.5 (13)
O11—Zr—O02^i^	72.91 (5)	O02—C04—C03	122.81 (17)
O11^i^—Zr—O02^i^	76.85 (5)	O02—C04—C05	118.06 (16)
O01^i^—Zr—O02	141.24 (5)	C03—C04—C05	119.11 (16)
O01—Zr—O02	75.41 (5)	C103—C102—C101	120.5 (2)
O11—Zr—O02	76.85 (5)	C103—C102—H102	119.8
O11^i^—Zr—O02	72.91 (5)	C101—C102—H102	119.8
O02^i^—Zr—O02	71.36 (7)	C14—C15—H15A	109.5
O01^i^—Zr—O12^i^	76.90 (5)	C14—C15—H15B	109.5
O01—Zr—O12^i^	72.46 (5)	H15A—C15—H15B	109.5
O11—Zr—O12^i^	140.87 (5)	C14—C15—H15C	109.5
O11^i^—Zr—O12^i^	75.27 (5)	H15A—C15—H15C	109.5
O02^i^—Zr—O12^i^	143.29 (5)	H15B—C15—H15C	109.5
O02—Zr—O12^i^	121.20 (5)	C104—C105—C100	121.7 (2)
O01^i^—Zr—O12	72.46 (5)	C104—C105—H105	119.1
O01—Zr—O12	76.90 (5)	C100—C105—H105	119.1
O11—Zr—O12	75.27 (5)	C103—C104—C105	120.2 (2)
O11^i^—Zr—O12	140.87 (5)	C103—C104—H104	119.9
O02^i^—Zr—O12	121.20 (5)	C105—C104—H104	119.9
O02—Zr—O12	143.29 (5)	C105—C100—C101	117.6 (2)
O12^i^—Zr—O12	71.35 (7)	C105—C100—C106	120.2 (2)
C12—O11—Zr	131.65 (12)	C101—C100—C106	122.2 (2)
C04—O02—Zr	134.45 (12)	C102—C101—C100	120.9 (2)
C02—O01—Zr	131.67 (12)	C102—C101—H101	119.5
C14—O12—Zr	134.72 (12)	C100—C101—H101	119.5
C12—C13—C14	120.47 (17)	C102—C103—C104	119.1 (2)
C12—C13—H13	119.8 (15)	C102—C103—H103	120.5
C14—C13—H13	119.4 (15)	C104—C103—H103	120.5
O01—C02—C03	127.88 (17)	C100—C106—H10A	109.5
O01—C02—C01	112.97 (16)	C100—C106—H10B	109.5
C03—C02—C01	119.15 (16)	H10A—C106—H10B	109.5
C04—C05—H05A	109.5	C100—C106—H10C	109.5
C04—C05—H05B	109.5	H10A—C106—H10C	109.5
H05A—C05—H05B	109.5	H10B—C106—H10C	109.5
C04—C05—H05C	109.5	C100—C106—H10D	109.5
H05A—C05—H05C	109.5	H10A—C106—H10D	141.1
H05B—C05—H05C	109.5	H10B—C106—H10D	56.3
O11—C12—C13	128.12 (17)	H10C—C106—H10D	56.3
O11—C12—C11	112.42 (15)	C100—C106—H10E	109.5
C13—C12—C11	119.40 (16)	H10A—C106—H10E	56.3
F01—C01—F02	107.98 (17)	H10B—C106—H10E	141.1
F01—C01—F03	106.60 (17)	H10C—C106—H10E	56.3
F02—C01—F03	106.60 (15)	H10D—C106—H10E	109.5
F01—C01—C02	111.26 (16)	C100—C106—H10F	109.5
F02—C01—C02	113.01 (16)	H10A—C106—H10F	56.3
F03—C01—C02	111.06 (16)	H10B—C106—H10F	56.3
F11—C11—F13	106.99 (15)	H10C—C106—H10F	141.1
F11—C11—F12	106.91 (15)	H10D—C106—H10F	109.5
F13—C11—F12	106.87 (15)	H10E—C106—H10F	109.5
F11—C11—C12	111.66 (15)		
			
O01^i^—Zr—O11—C12	−89.06 (15)	C14—C13—C12—O11	6.1 (3)
O01—Zr—O11—C12	53.60 (14)	C14—C13—C12—C11	−170.90 (17)
O11^i^—Zr—O11—C12	167.40 (15)	O01—C02—C01—F01	62.8 (2)
O02^i^—Zr—O11—C12	−155.08 (15)	C03—C02—C01—F01	−117.6 (2)
O02—Zr—O11—C12	130.68 (15)	O01—C02—C01—F02	−175.56 (16)
O12^i^—Zr—O11—C12	6.91 (18)	C03—C02—C01—F02	4.1 (3)
O12—Zr—O11—C12	−25.20 (14)	O01—C02—C01—F03	−55.8 (2)
O01^i^—Zr—O02—C04	−163.83 (14)	C03—C02—C01—F03	123.82 (19)
O01—Zr—O02—C04	28.28 (16)	O11—C12—C11—F11	54.2 (2)
O11—Zr—O02—C04	−55.32 (16)	C13—C12—C11—F11	−128.38 (18)
O11^i^—Zr—O02—C04	147.03 (17)	O11—C12—C11—F13	−65.2 (2)
O02^i^—Zr—O02—C04	−131.45 (18)	C13—C12—C11—F13	112.26 (19)
O12^i^—Zr—O02—C04	86.84 (17)	O11—C12—C11—F12	174.73 (15)
O12—Zr—O02—C04	−13.9 (2)	C13—C12—C11—F12	−7.8 (3)
O01^i^—Zr—O01—C02	167.64 (16)	Zr—O12—C14—C13	−17.5 (3)
O11—Zr—O01—C02	54.03 (15)	Zr—O12—C14—C15	165.24 (13)
O11^i^—Zr—O01—C02	−89.17 (15)	C12—C13—C14—O12	−6.0 (3)
O02^i^—Zr—O01—C02	6.93 (19)	C12—C13—C14—C15	171.22 (18)
O02—Zr—O01—C02	−24.70 (15)	O01—C02—C03—C04	7.0 (3)
O12^i^—Zr—O01—C02	−154.76 (16)	C01—C02—C03—C04	−172.56 (17)
O12—Zr—O01—C02	130.96 (16)	Zr—O02—C04—C03	−20.3 (3)
O01^i^—Zr—O12—C14	145.52 (17)	Zr—O02—C04—C05	161.44 (13)
O01—Zr—O12—C14	−57.13 (16)	C02—C03—C04—O02	−4.8 (3)
O11—Zr—O12—C14	26.49 (16)	C02—C03—C04—C05	173.47 (18)
O11^i^—Zr—O12—C14	−165.63 (15)	C100—C105—C104—C103	0.3 (4)
O02^i^—Zr—O12—C14	85.53 (17)	C104—C105—C100—C101	−1.1 (3)
O02—Zr—O12—C14	−15.3 (2)	C104—C105—C100—C106	178.4 (2)
O12^i^—Zr—O12—C14	−132.77 (19)	C103—C102—C101—C100	0.0 (4)
Zr—O01—C02—C03	15.3 (3)	C105—C100—C101—C102	1.0 (3)
Zr—O01—C02—C01	−165.07 (12)	C106—C100—C101—C102	−178.5 (2)
Zr—O11—C12—C13	17.1 (3)	C101—C102—C103—C104	−0.9 (4)
Zr—O11—C12—C11	−165.74 (11)	C105—C104—C103—C102	0.8 (4)

Symmetry codes: (i) −*x*+1, *y*, −*z*+3/2.

## References

[bb1] Allard, B. (1976). *J. Inorg. Nucl. Chem.* **38**, 2109–2115.

[bb2] Brandenburg, K. & Putz, H. (2005). *DIAMOND* Crystal Impact GbR, Bonn, Germany.

[bb3] Bruker (1998). *SADABS* Bruker AXS Inc., Madison, Wisconsin, USA.

[bb4] Bruker (2004). *SAINT-Plus* (including *XPREP*). Bruker AXS Inc., Madison, Wisconsin, USA.

[bb5] Bruker (2005). *APEX2* Bruker AXS Inc., Madison, Wisconsin, USA.

[bb6] Calderazzo, F., Englert, U., Maichle-Mössmer, C., Marchetti, F., Pampaloni, G., Petroni, D., Pinzino, C., Strähle, J. & Tripepi, G. (1998). *Inorg Chim. Acta*, **270**, 177–188.

[bb7] Clegg, W. (1987). *Acta Cryst.* C**43**, 789–791.

[bb8] Davis, A. R. & Einstein, F. W. B. (1978). *Acta Cryst.* B**34**, 2110–2115.

[bb9] Elder, M. (1969). *Inorg. Chem.* **8**, 2103–2109.

[bb10] Kurat’eva, N. V., Baidina, I. A., Stabnikov, O. A. & Igumenov, I. K. (2007). *J. Struct. Chem.* **48**, 513–522.

[bb11] Sheldrick, G. M. (2008). *Acta Cryst.* A**64**, 112–122. 10.1107/S0108767307043930 18156677

[bb12] Silverton, J. V. & Hoard, J. L. (1963). *Inorg. Chem.* **2**, 243–249.

[bb13] Viljoen, J. A., Roodt, A. & Muller, A. J. (2008). *Acta Cryst.* E**64**, m838–m839. 10.1107/S1600536808015237 PMC2961404 21202519

